# The interplay between regeneration and scavenging fluxes drives ocean iron cycling

**DOI:** 10.1038/s41467-019-12775-5

**Published:** 2019-10-31

**Authors:** Alessandro Tagliabue, Andrew R. Bowie, Timothy DeVries, Michael J. Ellwood, William M. Landing, Angela Milne, Daniel C. Ohnemus, Benjamin S. Twining, Philip W. Boyd

**Affiliations:** 1 School of Environmental Sciences, University of Liverpool, Liverpool, UK; 2 Institute for Marine and Antarctic Studies and Antarctic Climate and Ecosystems CRC, University of Tasmania, Hobsart, TAS Australia; 3 University of California Santa Barbara, Santa Barbara, CA USA; 4 Research School of Earth Sciences, Australian National University, Canberra, ACT Australia; 5 Florida State University, Tallahassee, FL USA; 6 University of Plymouth, Plymouth, UK; 7 Skidaway Institute of Oceanography, Savannah, GA USA; 8 Bigelow Laboratory for Ocean Science, East Boothbay, ME USA

**Keywords:** Element cycles, Marine chemistry

## Abstract

Despite recent advances in observational data coverage, quantitative constraints on how different physical and biogeochemical processes shape dissolved iron distributions remain elusive, lowering confidence in future projections for iron-limited regions. Here we show that dissolved iron is cycled rapidly in Pacific mode and intermediate water and accumulates at a rate controlled by the strongly opposing fluxes of regeneration and scavenging. Combining new data sets within a watermass framework shows that the multidecadal dissolved iron accumulation is much lower than expected from a meta-analysis of iron regeneration fluxes. This mismatch can only be reconciled by invoking significant rates of iron removal  to balance iron regeneration, which imply generation of authigenic particulate iron pools. Consequently, rapid internal cycling of iron, rather than its physical transport, is the main control on observed iron stocks within intermediate waters globally and upper ocean iron limitation will be strongly sensitive to subtle changes to the internal cycling balance.

## Abstract (web summary)

Iron is crucial for marine photosynthesis, but observational constraints on the magnitude of key iron cycle processes are lacking. Here the authors use a range of observational data sets to demonstrate that the balance between iron re-supply and removal in the subsurface controls upper ocean iron limitation.

## Introduction

Upper ocean primary production is limited by the availability of iron (Fe) over much of the ocean^1^. Even where nitrogen (N) and phosphorus (P) are the main limiting factors, Fe continues to play a key role by driving rates of N fixation^2^ and acquisition of dissolved organic P^3^. Fe limitation ultimately arises owing to a deficiency in the supply of Fe, relative to N and P^4^. Away from regions of dust deposition, the dominant component of Fe delivery, relative to N or P, is its relative concentration in thermocline waters^5^. This is particularly apparent across the south Pacific Ocean, where transport by sub-Antarctic mode water (SAMW) and Antarctic Intermediate water (AAIW) plays a key role in setting thermocline nutrient levels^6^. Accordingly, any fluctuations in the relative balance between Fe and major nutrients N and P in mode and intermediate waters in response to changes in climate will influence upper ocean Fe limitation and consequently modify global carbon and nitrogen cycles.

At present, there is low confidence in model projections of how modulations to climate will affect Fe supply to the upper ocean, as models generally show poor skill and substantial disagreement in their representation of the present-day ocean iron cycle. This lack of fundamental understanding of iron biogeochemistry is well illustrated by the order of magnitude inter-model variability in the residence time of iron in global models, despite aiming to reproduce the same ocean distributions from state of the art data sets^7^. Thus, despite a relatively long legacy of modelling the ocean iron cycle^8,9^, significant uncertainties in the magnitude of the major processes remain^1,10^. This means that although shifts in Fe inventories may indeed drive end-of-century trends in simulated productivity across much of the global ocean^11–15^, confidence in model projections is diminished by the lack of mechanistic constraints on their behaviour.

The ocean iron cycle is affected by an array of processes that interact together to set the dissolved iron concentrations in different parts of the ocean^16^. In the past decade, continental margins and hydrothermal vents have been acknowledged to augment dust deposition as important external iron sources^17,18^. Perhaps, most striking has been the recognition that the internal cycling of iron is typified by a range of biotic and abiotic transformations linked to Fe uptake (by both phytoplankton and bacteria), recycling, regeneration, scavenging and colloidal dynamics^10,19^. These processes act to shuttle dissolved iron between soluble and colloidal phases^20–22^ and drive transitions of particulate iron between biogenic, lithogenic and authigenic (i.e., the residual particulate Fe not accounted for by lithogenic and algal biogenic pools) components^23,24^. Despite these new insights, the relative magnitude of regeneration and scavenging, and crucially, the realised rate of net regeneration, is unknown at the spatial and temporal scales of mode and intermediate water transport. In part owing to these missing constraints, global ocean models used to assess the response of ocean ecology, biogeochemistry and the carbon cycle to environmental change are free to tune their internal iron cycle with residence times that vary from a few tens to a few hundreds of years^7^.

Newly expanded data sets of dissolved Fe (DFe) distributions from international ocean survey efforts within the GEOTRACES programme^25,26^ should facilitate model improvement, but only if quantitative insights into the governing processes can be determined. A particular challenge is to disentangle the balance between biogeochemical and physical processes in setting nutrient levels in the oceans’ interior. For example, total phosphate (PO_4_) at depth is made of up of two components: one associated with physical transport to depth (preformed PO_4_) and the other from the regeneration of P from organic matter degradation (regenerated PO_4_), which is quantified using apparent oxygen utilisation (AOU)^27,28^. A similar framework can be outlined for Fe, but Fe may be decoupled from P as it is affected by additional processes, such as extra Fe inputs onto intermediate water surfaces, unique regeneration of Fe, or Fe removal by scavenging^1,10,29^. Although scavenging of Fe will add complexity to the two-component model used for P, its magnitude remains an unknown quantity in observations. This lack of understanding is encapsulated by the evolving view of the ocean iron residence time from models and observations^7,30,31^.

Here we use observations to quantify the large scale modification of DFe, benchmarked to PO_4_, within the mode and intermediate waters of the south Pacific Ocean for the first time, using AOU to derive the role played by physics, regeneration and scavenging. We focus on mode and intermediate waters as they support the majority of global productivity through nutrient supply to surface waters^6^. This approach illuminates a highly dynamic interior ocean Fe cycle, within which the commonly measured DFe pool is only a small residual component. Consequently, additional measurements of the ocean iron cycle pools beyond DFe and in particular, Fe fluxes are necessary to better constrain internal cycling and reduce uncertainty in global climate model projections.

## Results

### Tracking South Pacific iron and phosphate accumulation

**Fig. 1:**
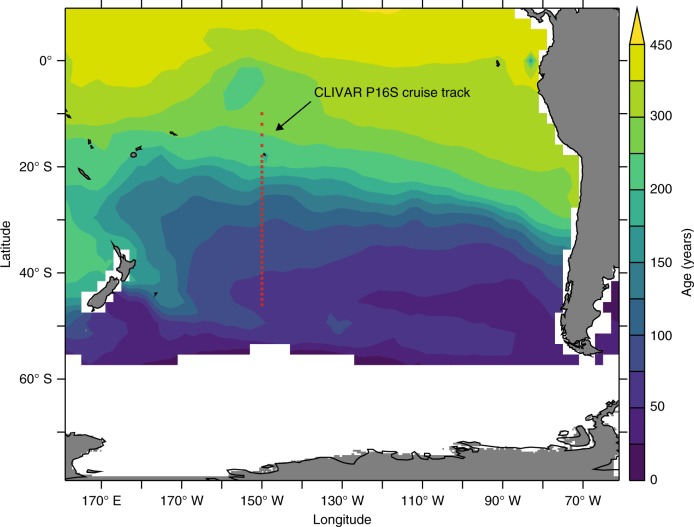
Study area. The southern part of the CLIVAR P16S line in the south Pacific Ocean, on a backdrop of water age (years) from the OCIM model averaged over the intermediate water isopycnal layers (*σ*_0_ = 26.8–27.2). The individual stations used in this analysis are marked with red crosses

Pacific Ocean SAMW and AAIW form in the southeast Pacific Ocean^32,33^ and their equatorward transport is well sampled by the southern part of the CLIVAR P16S cruise track along 150 °W (Fig. 1, Supplementary Fig. 1). We targeted the region 46°–10° S of the transect within a potential density window of 26.8–27.2 that broadly encompasses both SAMW and AAIW (hereafter, defined as intermediate water)^32,34^. In this density and latitude window, salinity was relatively well conserved at ~34.3–34.5 (indicating negligible mixing from multiple endmembers), and enough parallel observations of DFe, PO_4_ and oxygen were available (*n* = 89). As intermediate water moves equatorward, its core depth varies between 200 m and 800 m and AOU increases from 20 to 160 mmol m^−3^ as the constituents transported within the watermass, or delivered via sinking from above, undergo further remineralisation (Supplementary Fig. 1). Using an age tracer within a data-constrained ocean circulation inverse model (OCIM)^35^ that reproduces P16 salinity measurements, intermediate water in this density window aged by ~190 years (from 69 to 260 years) during this part of the P16S transect (Fig. 1, see also Supplementary Fig. 2).

**Fig. 2:**
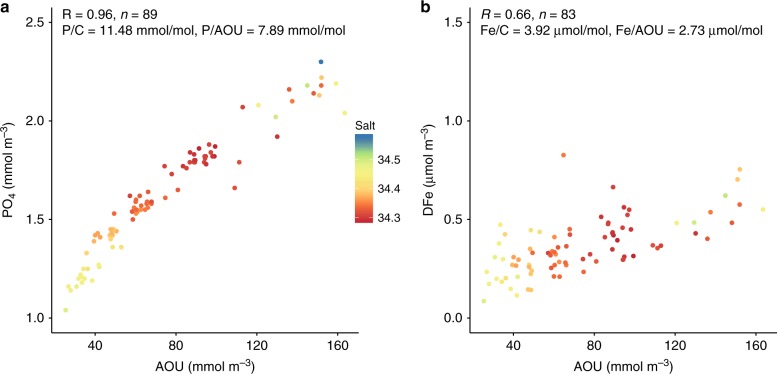
Linking phosphate and dissolved iron to apparent oxygen utilisation. Plots of PO_4_ (phosphate) and DFe (dissolved iron) observations against AOU (apparent oxygen utilisation) observations between the *σ*_0_ = 26.8–27.0 isopycnal layers along the P16S transect through the South Pacific Ocean, performed with a Type II regression. Salinity for each sample is coloured

As expected from our understanding of P biogeochemistry, PO_4_ is well correlated with AOU within the intermediate water layer (*R* = 0.96, Fig. 2a) and the slope of 11.48 ± 0.71 mmol P mol C^−1^ is very close to that expected from the organic matter content^36^. The intercept indicates a preformed PO_4_ concentration of 1.04 ± 0.04 mmol m^−3^ at the intermediate water outcrop in the Fe-limited Southern Ocean. More surprising is the broadly linear relationship between DFe and AOU within intermediate water (*R* = 0.66, Fig. 2b), with a slope of 3.92 ± 0.99 μmol Fe mol  C^−1^ and a preformed DFe concentration of 0.16 ± 0.06 μmol m^−3^. The Fe/C ratios estimated from the slope of the linear regression between Fe and AOU within AAIW are similar to those previously estimated from vertical profiles across the North Pacific Ocean^37,38^. However, the profile-based estimates cannot be used to quantify the accumulation of DFe since the zero AOU y-intercept that should represent the surface water outcrop of the isopycnal layer is instead the directly overlying surface water. This means that the values reported here are the first estimates of the temporal accumulation of DFe alongside concomitant oxygen consumption in intermediate waters. Indeed, we can use the watermass age estimate from OCIM to derive rates of accumulation of 6.75 μmol PO_4_ m^−3^ yr^−1^ and 2.34 nmol dFe m^−3^ yr^−1^ between 46 S and 10 S.

While the accumulation of PO_4_, relative to C, conforms to our prior understanding based on observations of P/C ratios from organic matter^36^, DFe accumulation appears very low, even for the Fe-poor South Pacific. Estimates of median phytoplankton Fe content from available synchrotron measurements (Table 1) range from 11.7 to 31.3 μmol Fe mol C^−1^, with an overall median value of ~15.7 μmol Fe mol C^−1^ typical of the South Pacific. This indicates that only around a quarter (25%) of phytoplankton Fe is accumulating as DFe in intermediate waters owing to regeneration. It is possible that living phytoplankton are not representative of the sinking detrital pool^39^, which could be addressed by examining Fe/C ratios within bulk particulate matter. However, total particulate Fe also includes relatively inert lithogenic Fe, which would then overestimate the labile (i.e., biotic) Fe content. To account for this, we estimated lithogenic Fe (see methods) from the only GEOTRACES particulate Fe data set from the Pacific Ocean (the zonal GP16 transect between Ecuador and Tahiti) using three different lithogenic models that account for a range of end-members from the Pacific basin^23,24,40^. After this correction, median non-lithogenic Fe/C ratios within all particulate samples, shallower than the lightest intermediate water isopycnal, range from 48.2 to 196.4 μmol Fe mol C^−1^, whereas the median P/C ratio is 12.73 mmol P mol C^−1^ (Table 1). This particulate analysis shows that the accumulation of dFe along the intermediate water pathway is only 2–8% of the non-lithogenic particulate Fe or ~25% of phytoplankton Fe from the upper ocean. In contrast, as expected from the two-component preformed-regenerated model of P cycling, almost all (90%) of the median particle P/C ratio (12.73 mmol P mol C−1) accumulates as PO_4_ (11.48 mmol P mol C−1) along the intermediate water pathway. This suggests that the simple two-component balance between regenerated and preformed pools that explains the internal cycling of PO_4_ is not applicable for Fe and the processes of subsurface DFe solubilisation during regeneration and removal via scavenging that control the net observable Fe remineralisation remain unconstrained.

**Table 1:** Meta-analysis of median and inter-quartile ranges (IQR) stoichiometric ratios from phytoplankton, particles (with different lithogenic corrections applied), sediment trap fluxes (with local estimates of lithogenic Fe or applying a conservative 80% lithogenic correction) and below mixed layer regeneration rates from process studies

		Fe/C		P/C	
	Detail	Median	IQR	Median	IQR
Phyto- plankton	South tropical Pacific	16.0	7.8–40.7		
	South Pacific^66^	15.3	9.7–26.5		
	Equatorial Pacific^67^	11.7	6.9–20.4		
	North Pacific	20.2	9.8–55.0		
	North Atlantic^68^	31.3	19.8–59.9		
Non-lithogenic Marine Particles^a^	Using Ti endmember	48.21	2.67–204.76	12.73	11.38–14.55
	Using Al endmember	196.4	105.5–396.7		
	Using Al/Ti endmember	103.35	56.69–175.83		
Export^b^	SAZ-Sense, Fe Cycle I and II sediment traps^44–46^	141.6	84.9-275.4	5.6	2.9-6.4

		Fe rate			
		Median	IQR		
Regeneration^c^	Experiments and budgets^45,47,48^	485.5	257.3-1113.3		

		Fe/C	Fe rate	P/C	P rate
Dissolved	Intermediate water (this study)	3.92 ± 0.99	2.34	11.48 ± 0.71	6.75

Median ratios and slopes are in units of μmol/mol (Fe/C) or mmol mol^−1^ (P/C), whereas rates are either nmol dFe m^−3^ yr^−1^ or μmol PO_4_ m^−3^ yr^−1^

^a^Particles collected from bottles during GEOTRACES GP16 voyage between Ecuador and Tahiti in the south Pacific above the intermediate water layer and west of station 23 to avoid influence of low-oxygen waters (*n* = 54) with three different lithogenic end members applied to remove lithogenic particulate Fe. Note P/C ratio results are unaffected by the choice of lithogenic end member

^b^Calculated non-lithogenic flux from sediment traps from the SAZ-Sense, Fe Cycle I and Fe Cycle II process studies, either by using local lithogenic corrections or a conservative estimate of 80% lithogenic Fe (*n* = 14 for Fe and 11 for P)

^c^Regeneration rates are compiled from all direct measurements of solubilisation of particles collected from below the mixed layer and iron budget calculations of iron regeneration (*n* = 6)

### Controls on dissolved iron accumulation in intermediate waters

There are three main hypotheses to explain the mismatch between accumulation of DFe and the magnitude of phytoplankton and particulate Fe stocks that fuel DFe replenishment. The first hypothesis states that particulate Fe is not exported from the surface ocean and is instead retained in the zone shallower than the upper bound of intermediate waters. The second hypothesis states that particulate Fe is exported out of the upper ocean but is not regenerated. Finally, the third hypothesis states that ample Fe is exported and regenerated, but strong removal (e.g. by scavenging) of regenerated Fe leads to minor accumulation of DFe.

The first hypothesis can be rejected as although recycling of Fe in the upper ocean is significant, ample particulate Fe is exported from the surface ocean. Significant recycling of Fe in the upper mixed layer has been demonstrated from a variety of field studies and budget calculations^5,10,19,41–43^, which indicate substantial turnover of the particulate Fe pool. Measurements of particulate Fe exported from the upper ocean from trace metal clean sediment traps are very rare, but, where available, also support substantial export of particulate Fe. Sinking particulate Fe flux data from the SAZ-Sense and Fe Cycle I and II (at ~100 m depth and either directly accounting for lithogenic Fe or taking a conservative 80% estimate of the lithogenic fraction^44^) results in non-lithogenic Fe/C export ratios of between 30 and 400 μmol Fe mol C^−1^ and P/C export ratios of ~6–8.5 mmol P mol C^−1^ across all data^44–46^ (all broadly similar to those from non-lithogenic mixed layer particles, Table 1). Median values from both data sets produce flux ratios of 141.6 μmol Fe mol C^−1^ and 5.6 mmol P mol C^−1^, compared with accumulation ratios of 3.9 μmol Fe mol C^−1^ and 11.5 mmol P mol C^−1^ (Table 1). Thus, despite intense recycling in the surface mixed layer, export fluxes of non-lithogenic Fe out of the base of the surface mixed layer are significant relative to the accumulation of DFe observed during regeneration along intermediate water pathways in the oceans’ interior (Table 1), leading us to reject hypothesis one.

The second hypothesis can be rejected in light of previous assessments of solubilisation of Fe from particles below the mixed layer (at between 100 and 200 m) through a set of experiments that incubated a subsurface particle assemblage resuspended from McLane pump 142 mm filters and monitored the release of DFe, as well as by iron budget calculations. These estimates are also sparse, but for two distinct field experiments, dFe release rates range between 511 and 1314, and 120 and 460 nmol m^−3^ yr^−1^ from particles from below the mixed layer^47,48^. Budget based calculations are similar, producing subsurface dFe regeneration rates of 190–263 nmol m^−3^ yr^−1^ at 100 m^45^. Across all estimates we find a median of 485 nmol m^−3^ yr^−1^, two orders of magnitude greater than the dFe accumulation rate of ~ 2 nmol m^−3^ yr^−1^ we find within intermediate water (Table 1). These regeneration rates are clearly substantial, and we are required to reject hypothesis two.

Based on our rejection of the first two hypotheses, we are required to invoke a significant loss of regenerated Fe when considering hypothesis three. This would reconcile the low rates of dFe accumulation within intermediate waters with the significant export of non-lithogenic Fe and large rates of dFe solubilisation from sinking particles. The potential role of the removal of regenerated algal biogenic Fe has been previously observed using synchrotron-mapping of particles derived from sediment traps^49^ and would also explain observations of an increasing association of sinking non-lithogenic particulate Fe with authigenic phases in deep-moored sediment traps (between 500, 1500 and 3200 m) in the Atlantic^50^. For the Pacific, we calculate that 20–40% of the particulate Fe within the intermediate water in the western portion of the GP16 Pacific section cannot be accounted for by the sum of lithogenic and algal biogenic components. This implies a non-negligible authigenic particulate Fe component that would be consistent with removal of regenerated Fe by scavenging.

## Discussion

Our results point to continual removal of regenerated iron, resulting in only a small accumulation of DFe within intermediate waters. The combination of the constant rain of new material and the disaggregation of sinking particles in the ocean interior may be able to maintain scavenging of released Fe as the increasing surface area:volume ratio provides new surfaces for scavenging. Indeed, the increase in the flux of small particles (11–64 μm, equivalent spherical diameter, ESD) off Bermuda, and the concomitant opposite trend for large (>64 μm ESD) particles at depth^51^, highlights the important role this may play in producing small particles. Similarly, number spectrum analyses (using underwater video cameras) across the upper 200 m of the water column in the S. Pacific Gyre reveal much higher abundances of small particles than larger ones^52^. As scavenging of trace metals like Fe is highly dependent on surface area^53–55^, these particle disaggregation/fragmentation processes can catalyse further scavenging of the dFe released by regeneration. Scavenging of regenerated Fe into authigenic phases may also enhance particle sinking rates by increasing the specific gravity of particles (as noted for lithogenic Fe^56^). These abiotic processes may act in concert with the removal of solubilised Fe by heterotrophic bacteria operating within particle microenvironments^57,58^. If we take our median estimated regeneration rate of dFe and the estimated accumulation rate of dFe (Table 1), and then combining these with a typical intermediate water layer thickness of 300 m at 10 °S, requires net downward removal fluxes of ~0.39 μmol m^−2^ d^−1^. Although these fluxes would be inconspicuous in the measurements spanning ~0.4–10 μmol m^−2^ d^−1^ from trace metal clean sediment traps^44,45^, they are crucial in shaping the basin scale internal cycling of dFe in intermediate water layers.

We observe a small, but significant, accumulation of DFe with time (Fig. 2b), suggesting that the net regeneration quantified by the slope of the DFe versus AOU relationship integrates the balance between regeneration and scavenging fluxes. Observed concentrations of weak Fe-binding ligands are typically well in excess of DFe levels, which would imply an ample capacity to stabilise regenerated Fe at much higher levels^59–62^ and is not in agreement with our analysis. However, the muted increase in DFe we observe is very consistent with the apparent saturation of strong Fe-binding ligands by DFe pools in the south Pacific Ocean^60^. This would imply that strong Fe-binding ligands, rather than their weaker counterparts, may play a key role in shaping the dissolved Fe distribution in the oceans’ interior. An additional role may be played by the interplay between soluble and colloidal iron pools, which can also be part of the ligand pools^20–22^ and in the future it may be useful to compare the net regeneration derived from the DFe-AOU slope to observations of colloidal iron. Finally, we emphasise that the putative production of authigenic Fe from the DFe solubilised during regeneration, that we term here as scavenging, might not occur in the water column, but instead within particles and their associated microenvironments^57,58^ in a manner disconnected from the wider water column ligand pool.

**Fig. 3:**
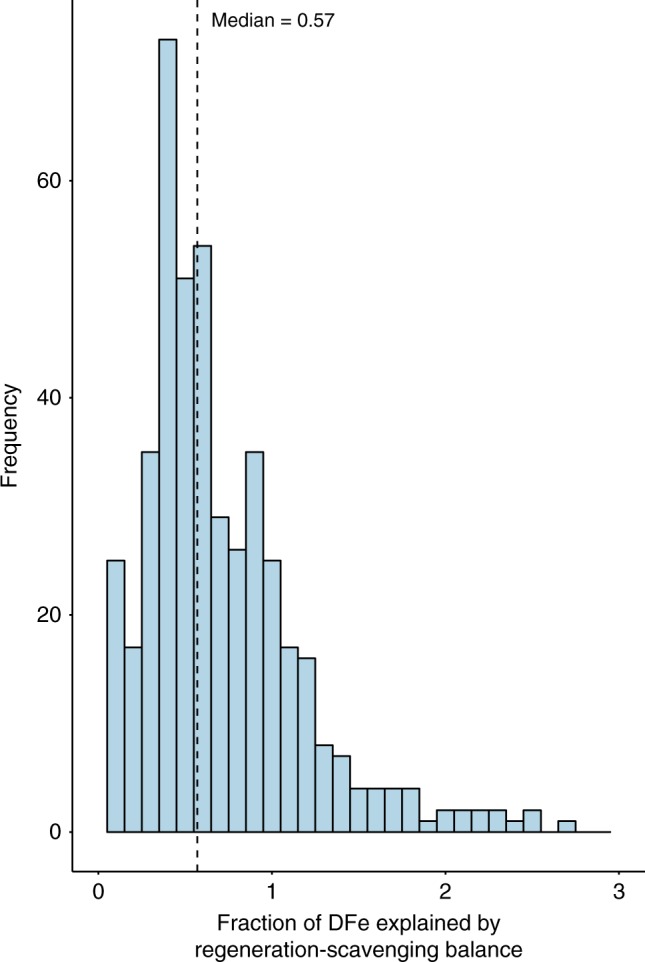
Quantifying the origins of dissolved iron in the GEOTRACES IDP2017 dataset. The fraction of the dissolved iron concentration from the IDP2017 explained by the regeneration—scavenging balance between the *σ*_0_ = 26.8–27.0 isopycnal layers is quantified here. The magnitude of the regeneration—scavenging balance (Fe_REG'_, mol m^−3^) can be derived by using the slope of the apparent oxygen utilisation—dissolved iron relationship from the P16 transect (2.7 μmol dissolved iron per mol apparent oxygen utilisation) and the independent apparent oxygen utilisation and dissolved iron data sets from the GEOTRACES IDP2017. The estimated net regeneration of dissolved iron (Fe_REG'_) is then divided by the observed total dissolved iron from the IDP2017 to quantify the fraction explained by the regeneration—scavenging balance. The median value of 0.57 is indicated with a vertical dashed line. This indicates that over half of the observed dissolved iron is explained by the regeneration—scavenging balance, with less than half explained by ocean physical transport

The DFe-AOU slope of 2.7 μmol DFe mol AOU^−1^ from our analysis (Fig. 2b) permits us to examine what proportion of the DFe pool might be controlled by the net interplay between regeneration and scavenging (termed ‘internal cycling’ hereon). It is well understood that roughly two-thirds of the interior PO_4_ signal is preformed (controlled by physical transport), with the remaining one-third due to regeneration^27,28^. In contrast to PO_4_, the proportion of the DFe pool controlled by internal cycling in intermediate waters (within the 26.8–27.2 isopycnal layer) across the entire available GEOTRACES data set^26^ of DFe and AOU has a median value of 0.57 (Fig. 3). This implies that over half of the DFe concentration in intermediate water is in fact set by internal cycling (i.e., the interplay between regeneration and scavenging), with the remainder controlled by physical transport of preformed DFe (either from the ocean surface or laterally). The stronger role played by preformed PO_4_ than preformed DFe arises owing to the higher unused PO_4_ levels in the, typically Fe-limited, watermass outcrop regions. Thus, because DFe is drawn down to very low levels in regions of intermediate water formation, internal cycling has a larger imprint on the interior DFe concentrations across much of the globe than for PO_4_. This view agrees with the lack of clear watermass signals in large scale ocean DFe sections^63^ and is at odds with simulations from iron models with a dominant physically transported component.

Overall, the strong mismatch we find between the internal basin scale Fe cycle fluxes and the residual DFe pool that accumulates from their interplay explains why Fe models can produce such divergent residence times while trying to reproduce the same dFe data sets. Our analysis finds DFe to be rapidly cycled by Fe supply and removal processes, which supports those models parameterised with short residence times. The net regeneration that shapes the multi-decade accumulation of DFe in intermediate waters is likely controlled by some combination of strong iron-binding ligands, colloidal dynamics, bacterial and authigenic iron pools. Because of the dominance of internal cycling, the concentration of Fe (relative to major nutrients N and P) and hence upper ocean iron limitation, will be strongly sensitive to small changes in the gross fluxes that govern the net regeneration of Fe. For instance, the Fe content of upper ocean phytoplankton is highly variable and fluctuations due to changing iron supply or phytoplankton species composition will affect the gross regeneration fluxes. Alternatively, biological and chemical transformations of particles, strong iron-binding ligands, bacterial demand and/or iron speciation will modify gross scavenging rates. Both these examples would change the net regeneration rate and hence the relative supply of Fe to the upper ocean microbes. Our isopycnal framework also provides a mechanistic methodology to assess ocean biogeochemical models more rigorously in future model evaluation efforts. A new generation of in situ processes studies^1^, tracking the evolution of Fe biogeochemistry by measuring both fluxes and particulate and dissolved Fe pools within a coherent physical framework would offer the potential to further constrain the internal cycling mechanisms for inclusion into global biogeochemical models. This improved mechanistic understanding of the ocean Fe cycle is required to reduce uncertainties in how changes in climate will affect surface ocean Fe limitation of primary productivity over much of the global ocean.

## Methods

### Field sampling and data processing

Sampling along the CLIVAR P16 section was conducted during two cruises, from Tahiti to Kodiak, Alaska aboard the R/V Thomas Thompson (15th February—25th March 2006;  P16N), and from Tahiti to Antarctica aboard the R/V Roger Revelle (9th January–22 February 2005; P16S). Samples for dFe were analysed following previously published protocols^64^. In brief, 15 mL aliquots of acidified (0.024 m, HCl) sample were spiked with 100 µL of an ^57^Fe isotope-enriched solution (Fe concentration of 177 nm) and UV-oxidised (>1 h). After cooling overnight, samples were buffered with ammonium acetate to pH 6.4 ± 0.2 prior to being passed through a column packed with Toyopearl AF-Chelate-65 M. Extracted Fe was subsequently eluted with 1 m HNO_3_ into 1 mL aliquots and analysed by high resolution-inductively coupled plasma-mass spectrometry (Thermo Finnigan Element 1). dFe concentrations were quantified using a standard isotope dilution equation. The analytical limit of detection (LOD; 3xSD of blank) averaged 0.019 nm (*n* = 20) during the analysis period, while the procedural LOD (based on 3xSD of replicate analysis of SAFe S1) averaged 0.034 nm (*n* = 29). Accuracy and precision were assessed through the replicate extraction and analysis of SAFE and GEOTRACES seawater reference materials^64^. Typical within run precision averaged 2.2% (1RSD, *n* = 27) at iron concentrations ~1 nm and 11.8% (1RSD, *n* = 29) at lower iron concentrations (~0.1 nm). AOU was calculated from oxygen saturation (derived using temperature and salinity). DFe, PO_4_ and AOU were binned within the intermediate water density layers (28.6–27.2) and between latitudes of 46 °S and 10 °S using P16S data. Statistics were performed using Type II regressions via the R package ‘lmodel2’. The net regeneration (Fe_REG_’) that results from the near-balance between regeneration and scavenging is derived by combining the Fe/AOU slope from the P16 with AOU using oxygen, temperature, salinity and DFe data from the indepedent GEOTRACES IDP2017^26^ between the 26.8 and 27.2 isopycnal layers that represent intermediate water. Field data from the P16 voyage is available from BCO-DMO^65^.

### Corrections for lithogenic and algal biogenic Fe

Presuming that total particulate Fe in any sample is the sum of algal biogenic (PFeBio, P-associated), lithogenic (PFeLith, Al- or Ti-associated), and authigenic sub-fractions, we estimate the authigenic, or scavenged, Fe (PFeAuth) by sequentially subtracting estimated lithogenic (PFeLitho), non-lithogenic (PFeNonLitho) and authigenic (PFeAuth) fractions via the following three balances: PFeTotal = PFeLitho + PFeBio + PFeAuth, PFeNonLitho = PFeTotal − PFeLitho and PFeAuth = PFeNonLitho − PFeBio. In this study, we based lithogenic Fe corrections on two assumptions: (1) lithogenic material in the ocean is ultimately derived from a crustal source(s) with estimable, fixed composition(s), and (2) lithogenic particles are refractory, meaning that elemental exchange with dissolved or other particulate pools during their marine residence times (weeks to years)^24^ does not significantly alter their composition. To estimate and correct for lithogenic Fe we quantified the number and composition of potential lithogenic endmembers. The ratios of Al, Ti and Th were used to address the compositional gradients of lithogenic particles in the GP16 transect and estimate the fractional composition of each endmember (see Supplementary Note and Supplementary Figs. 3 and 4). We then correct for lithogenic Fe using Fe/Al or Fe/Ti ratio(s) from one or more endmember(s) in turn for a total of three lithogenic Fe estimates. Finally, algal biogenic Fe (PFeBio), is derived from particulate phosphorus concentrations and estimates of the algal biogenic Fe/P ratio, with PFeAuth emering as a residual pool. This analysis is performed using data from the south Pacific GP16 section from the GEOTRACES IDP2017^26^.

## Supplementary information

Supplementary Information

## Data Availability

All the data used in this research are freely available and may be downloaded through the links detailed in the Methods section.
