# Supplementary material for: The interplay between regeneration and scavenging fluxes drives ocean iron cycling
Source: Nat Commun. 2019 Oct 31;10:4960. doi: 10.1038/s41467-019-12775-5 (PMC6823497; doi:10.1038/s41467-019-12775-5)
Supplement: Supplementary file 1 — Supplementary Information [file 41467_2019_12775_MOESM1_ESM.pdf]

Supplementary information for: The interplay between regeneration and scavenging fluxes drives ocean iron cycling

Alessandro Tagliabue[1*], Andrew R. Bowie[2], Timothy DeVries[3], Michael J. Ellwood[4], William M. Landing[5], Angela Milne[5,6], Daniel C. Ohnemus[7], Benjamin S. Twining[8] and Philip W. Boyd[2]

Supplementary Note: Examining Ti-normalized lithogenic ratios (Al/Ti, Th/Ti) in bulk particles from across the transect, we see considerable spread compared to planet-wide lithogenic rock composition (GEOROC database samples, Supplementary Figure 3a) suggesting more than one lithogenic input to the basin. Broadly, lithogenic ratios vary from east (nearer South American continental dust, shelf, and/or riverine inputs; higher Th/Ti, Al/Ti) to west (nearer ocean island basalts and/or longer-distance Asian or Australian aeolian influences; lower Th/Ti, Al/Ti). While these ratios cannot unambiguously identify the exact end-members (Supplementary Figure 3bc) they nevertheless suggest that broad nearshore-offshore lithogenic compositional gradients do exist. Use of a single fixed Fe/Al or Fe/Ti ratio (Fe/X) from either extreme of the transect to calculate non-lithogenic Fe in any given sample:

Fe_NonLith_1EM = FeTotal – (Fe/X)_1EM * X

would generally over- or under-estimate lithogenic Fe for large portions of the transect [Supplementary Figure 4a, horizontal lines]. To potentially improve upon, but to also compare to the single end-member approach, we empirically chose two end-members from the eastern and western extremes of the transect, then estimated the lithogenic mixture in each sample presuming linear mixing between the two: a linear fit between the end-member compositions serves as the compositional continuum, and samples off the line are mapped to it via lines of opposite slope. The two end-member approach results in transect-wide lithogenic compositional gradients that are consistent with spatial trends in crustal samples from the GEOROC dataset [Supplementary Figure 3bc]. Examining trends in Fe/Al and Fe/Ti ratios as a function of fractional end-member lithogenic composition [Supplementary Figure 4a] we find similarly consistent trends with GEOROC compositional ratios (Supplementary Figure 4b; elevated Fe/Ti and lower Fe/Al nearshore), suggesting

the approach captures significant amounts of lithogenic Fe variability as well. Using Fe/Ti or Fe/Al ratios in litho-rich samples at the east/west (nearshore/offshore) extremes of the transect as Fe-compositional end-members, we then correct total particulate Fe to “non-lithogenic” particulate Fe in each sample:

Fe_NonLitho_2EM = FeTotal – X*([Fe/X_NS] * FractLithoNS + [Fe/X_OS]*(1-FractLithoNS))

Supplementary Figures:

Supplementary Figure 1: Properties measured on the P16 cruise between 40S and 10S: a) potential density anomaly, b) phosphate, c) oxygen, d) apparent oxygen utilisation and e) dissolved iron. Contour plots made using Ocean Data View[1] with a weighted average gridding setting of 35 permille scale lengths for X and Y.

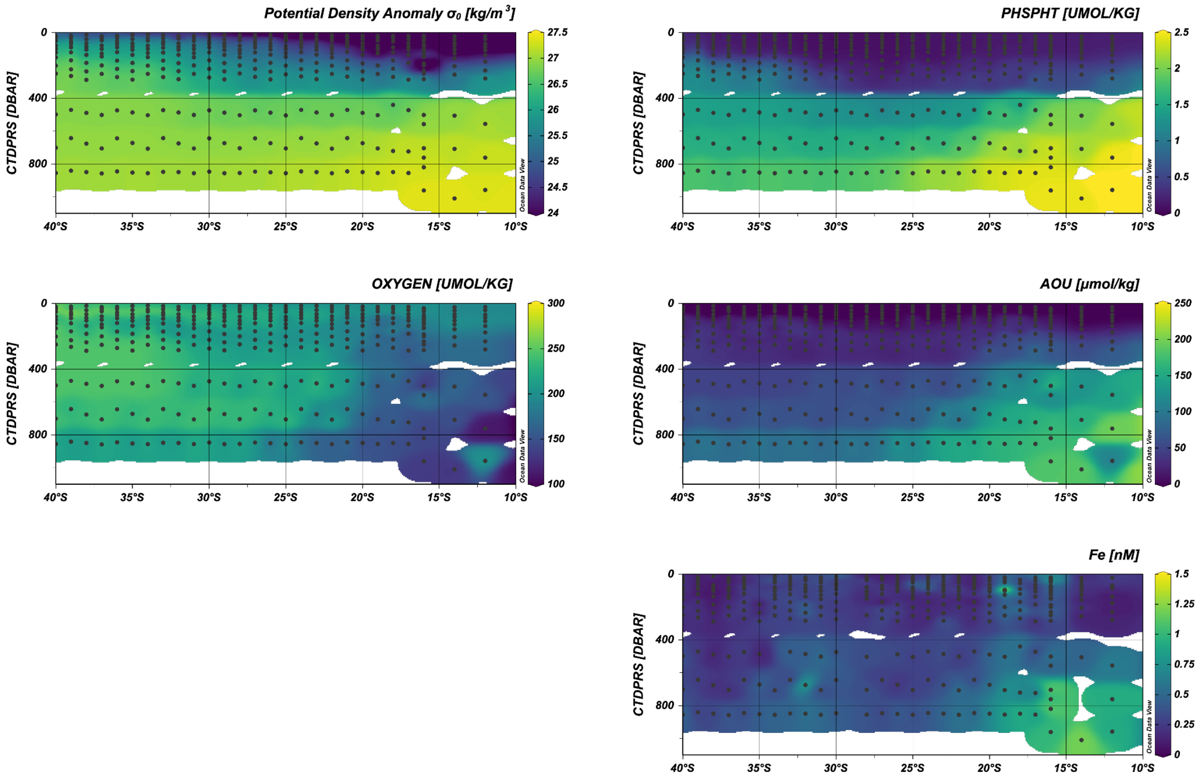

Supplementary Figure 2: Evaluation of OCIM salinity[2] against in situ salinity for P16 as a function of latitude. Values are isolated from the same station locations as the P16, from the σ_0=26.8-27.2 isopycnal window:

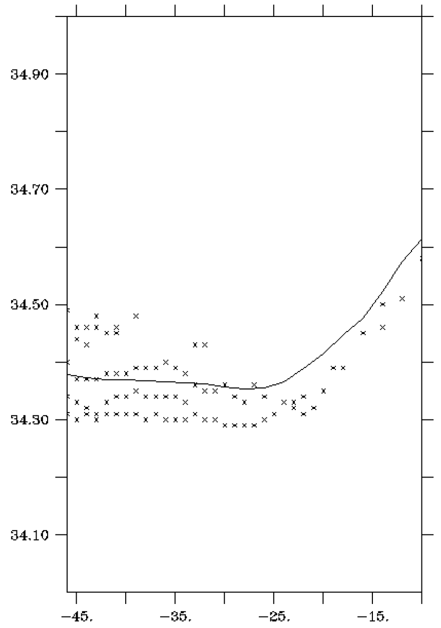

Supplementary Figure 3 A: The global GEOROC database contains >214,000 igneous, whole-rock, and mineral samples from continental and oceanic sources for which Al, Ti, and Th content has been measured. GEOROC sample compositions are mapped onto a 0.1 x 0.1 grid defined by Th/Ti and Al/Ti ratios in log-space to visualize the global lithogenic compositional distributions for these ratios (contours). Most GP16 water column samples (circles) overlap with the GEOROC database, confirming the strong lithogenic character of these three elements in marine particles. However, marine samples from across the 8000+ km transect also show significant spread in composition compared to the global database, suggesting that use of a single lithogenic compositional end-member (EM) is insufficient to capture the observed lithogenic variability. We empirically chose two end-members (stars) from lithogenic-rich samples at east/west extremes of the transect to represent near-shore and offshore-like lithogenic particles, consistent with near-shore/off-shore trends in the ratios from GEOROC samples (**B and C:** maps; cruise track in black). For each GP16 sample, the estimated fraction nearshore-like is shown (circle colors in **A**).

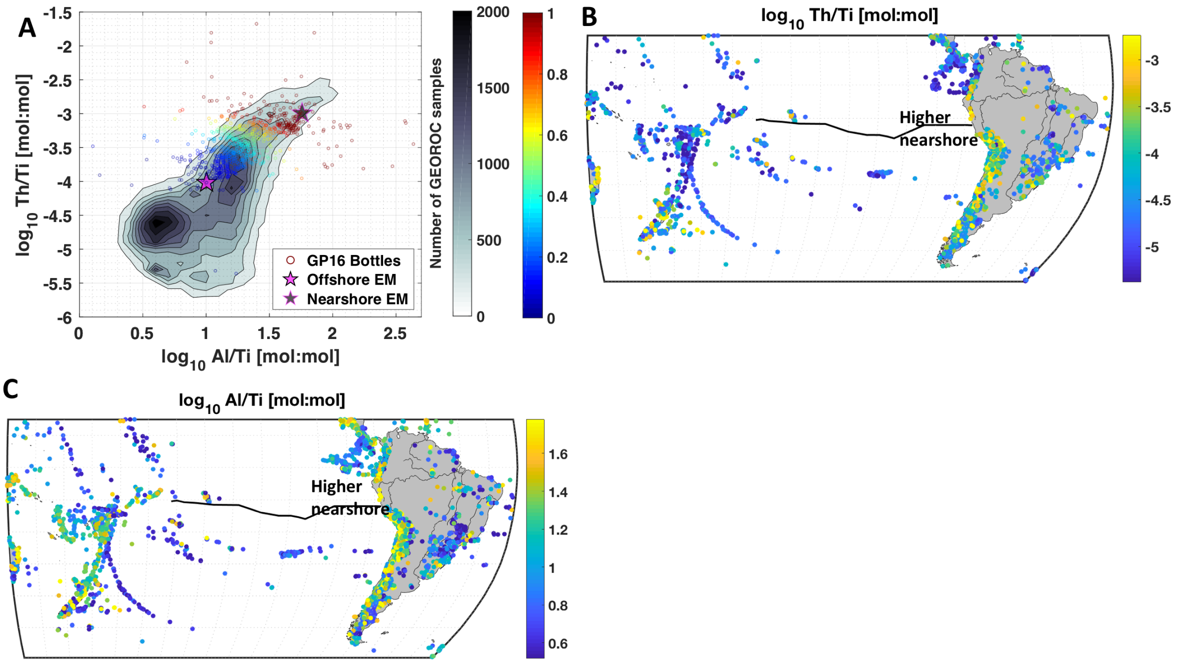

Supplementary Figure 4. A: After estimating fractional nearshore-like lithogenic content in GP16 bottle samples (individual datapoints, colored by broad water column sampling regime) using Al/Ti and Th/Ti ratios in two end-members (x-axes), nearshore/offshore trends in the "baseline" Fe/Ti and Fe/Al content of water column particles (y-axes) are also observed: broadly higher Fe/Ti nearshore, lower Fe/Al nearshore. While unable to unambigiously identify the exact end-members, these trends are consistent with transect-wide trends in Fe/Ti and Fe/Al ratios in crustal samples from the GEOROC database **(B)**. Use of a single near-shore-like lithogenic end-member (flat horizontal trendlines in D) to estimate lithogenic Fe would tend to over-estimate (Fe/Ti; **A,** upper panel) or under-estimate (Fe/Al; **A**, lower panel) lithogenic Fe in many offshore water column samples. Use of a two end-member approach (dotted trendlines) that varies between a nearshore-like and a Ti-richer/Al-poorer offshore end-member better fits the baseline Fe-content of particles for both Ti- and Al-normalized ratios.

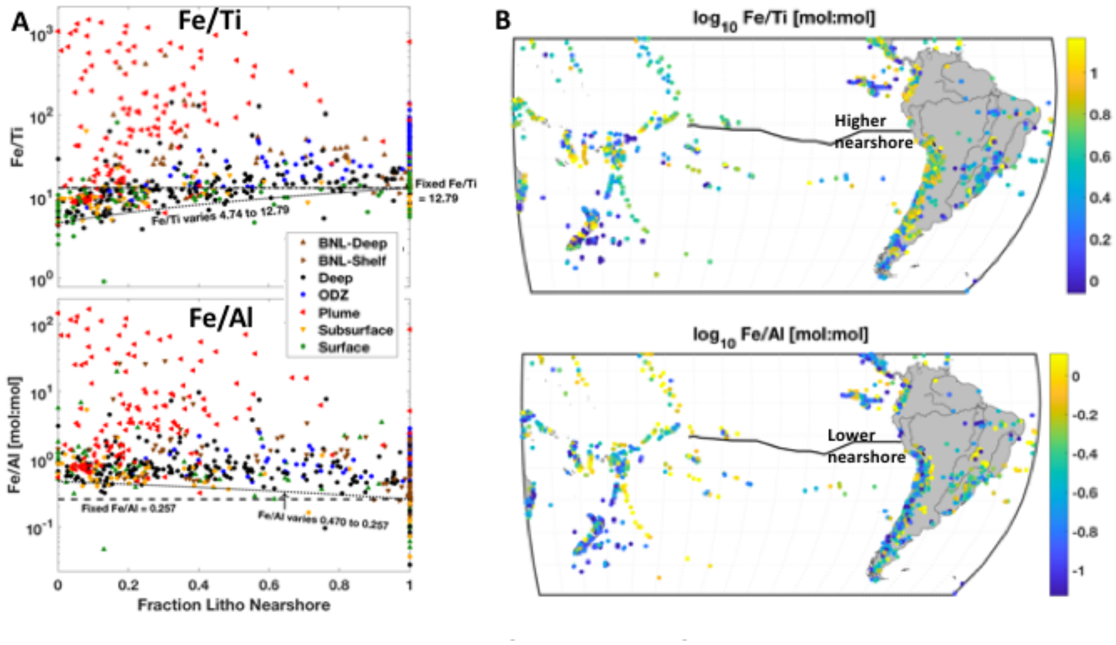

Supplementary References:

1 Schlitzer, R. Ocean Data View. https://odv.awi.de/ (2018).

2 DeVries, T. The oceanic anthropogenic CO2sink: Storage, air-sea fluxes, and transports over the industrial era. *Global Biogeochemical Cycles* **28**, 631-647, doi:10.1002/2013gb004739 (2014).
